# Peritonsillitis in primary care: patient characteristics and care pathways – a population-based registry study

**DOI:** 10.1186/s12875-026-03486-0

**Published:** 2026-08-07

**Authors:** Jon Pallon, Katarina Hedin

**Affiliations:** 1 Department of Clinical Sciences in Malmö, Family Medicine, Lund University, Malmö, Sweden; 2 Department of Research and Development, Region Kronoberg, Växjö, Sweden; 3 Futurum, Region Jönköping County, Jönköping, Sweden; 4 Department of Health, Medicine and Caring Sciences, Linköping University, Linköping, Sweden; 5 Clinical Research Centre, Institutionskansliet för kliniska vetenskaper, Box 50332, Malmö, 202 13 Sweden

**Keywords:** Peritonsillitis, Peritonsillar abscess, Primary care, Incidence, Registry study, Antibiotic prescribing, Referral pathways

## Abstract

**Background:**

Peritonsillitis, including peritonsillar abscess and cellulitis, is a common deep neck infection often considered a complication of pharyngotonsillitis. However, population-based data on patient characteristics, clinical presentation, and care pathways are limited.

**Methods:**

We conducted a population-based registry study in Region Kronoberg, Sweden (2012–2016), including all episodes of peritonsillitis and pharyngotonsillitis diagnosed in primary health care (PHC) and hospitals. New episodes were defined as diagnoses separated by ≥ 30 days. Patient characteristics, recent pharyngotonsillitis, and referral pathways between PHC and specialist care were described. Incidence rates and temporal trends were estimated to provide population context.

**Results:**

A total of 607 peritonsillitis episodes were identified in 555 patients. Incidence peaked in late adolescence and young adulthood, remained low in children and older adults, and showed no sex differences within age groups. Only 23% of episodes were preceded by pharyngotonsillitis within 30 days (median interval: 2 days), increasing to 29% when restricting the analysis to cases confirmed in the ear, nose, and throat (ENT) department. Most patients (74%) were first diagnosed in PHC, and 71% of these were subsequently managed in the ENT department. Overall, 85% of all episodes involved initial PHC management. Incidence of peritonsillitis declined over time in parallel with pharyngotonsillitis at the population level.

**Conclusions:**

Peritonsillitis predominantly affects adolescents and young adults and commonly presents without a recent documented sore throat. PHC plays a central role in initial assessment and referral, with most patients subsequently managed in specialist care. These findings provide population-level insights into patient characteristics and care pathways, with implications for early recognition and management in PHC.

## Introduction

Peritonsillitis is an infectious inflammation of the peritonsillar space, located between the tonsil and the adjacent pharyngeal muscles. It represents the most common deep neck space infection and encompasses a spectrum ranging from localized inflammation (peritonsillar cellulitis) to the formation of a purulent collection (peritonsillar abscess, ”quinsy”) which requires antibiotic therapy and, when an abscess is present, surgical drainage. The estimated incidence of peritonsillar abscess ranges from 19 to 42 cases per 100,000 inhabitants per year and appears to be increasing over time [1–3]. There has also been observed a sudden rise in incidence after the Covid 19-pandemic, suggesting an immunity debt [4]. Although individuals of all ages may be affected, peritonsillitis occurs most frequently among adolescents and young adults [1, 5]. Additional risk factors include male sex, smoking, immunosuppression, periodontal disease, and recent antibiotic use [1, 5, 6]. In contrast, clinical features and commonly used assessment tools at presentation – such as the FeverPAIN and Centor scoring systems, as well as laboratory markers including C-reactive protein and white blood cell count – show limited diagnostic utility [7].

The microbial etiology of peritonsillitis remains a subject of debate [1, 8]. Due to the high density of commensal bacteria in the oropharynx, it is often difficult to determine whether an isolated species is truly pathogenic. Both monomicrobial and polymicrobial infections have been reported [8]. Cultures obtained from abscess aspirates commonly yield organisms from the normal oropharyngeal flora, including both aerobic and anaerobic species [8]. The most frequently identified pathogens include *Streptococcus pyogenes* (Group A Streptococcus, GAS), *Fusobacterium necrophorum*, *Fusobacterium nucleatum*, members of the *Streptococcus anginosus* group, and anaerobes (*Prevotella*, *Porphyromonas*) [1, 8–10]. Clinical observations suggest that polymicrobial infections may be associated with increased severity, possibly due to synergistic interactions between bacterial species [5]. Viral involvement has not been demonstrated [11].

The pathogenesis of peritonsillitis also remains uncertain [5, 12]. A widely accepted theory proposes that the infection develops as a complication of acute pharyngotonsillitis, with bacteria penetrating the tonsillar capsule and spreading into the peritonsillar space [5, 12]. However, not all cases are preceded by pharyngotonsillitis. Retrospective studies suggest that this is the case in only about half of the patients [12], although reported proportions vary widely from 4% to 79% [3, 12, 13]. Furthermore, antibiotic treatment of pharyngotonsillitis appears to provide only weak to modest protection [13–17]. An alternative hypothesis suggests that the infection may originate in the minor salivary glands (Weber’s glands), located near the tonsils and involved in mucosal cleansing [5, 18–22]. Recurrent or chronic infections could lead to fibrotic changes and glandular obstruction, thereby predisposing individuals to subsequent infection. Ultimately, both hypotheses may be true and complementary [12].

Although management of peritonsillar abscess typically occurs in hospital settings, most often within otorhinolaryngology (ENT) departments, patients frequently present initially in primary health care (PHC) [3, 14]. Nevertheless, little is known about how patients with peritonsillar cellulitis or abscess are managed in PHC, or how they transition between PHC and ENT services. Improved knowledge of the characteristics and care pathways of patients with peritonsillitis could help PHC clinicians identify at-risk individuals and optimize early management. Furthermore, data on preceding episodes of pharyngotonsillitis may offer insights into disease pathogenesis and inform antibiotic prescribing strategies in PHC.

The aim of this population-based registry study was to (1) describe the demographic, epidemiological, and laboratory characteristics of patients diagnosed with peritonsillitis, and (2) analyse care pathways and healthcare utilization between PHC and ENT services in Region Kronoberg, Sweden (2012–2016).

## Materials and methods

This study presents a new analysis of routine healthcare data collected from 2012 to 2016, originally extracted from the regional data warehouse to investigate the relationship between bacterial aetiology, antibiotic prescribing, and clinical course in patients diagnosed with pharyngotonsillitis in PHC [21]. The same dataset was subsequently used to examine the use of point-of-care tests in diagnosing pharyngotonsillitis [22]. The findings are reported in accordance with the RECORD statement [23].

### Study population and setting

This study was conducted in Region Kronoberg, located in southern Sweden, with a median population of 189,634 during the study period. The Swedish healthcare system is publicly funded through taxes and is accessible to all residents as part of the country’s welfare system. The system includes approximately 1,200 primary health care centrals (PHCCs) across the nation, which serve as the first point of contact for patients and can refer individuals to hospitals when necessary. The vast majority of respiratory infections are initially assessed at PHCCs. During the study period, 31 out of the 34 PHCCs in Region Kronoberg participated in the study. Additionally, the Region’s two out-of-hours centers, operating from 17:00 to 21:00, as well as the Region’s two hospitals, were involved. During the study period (2012–2016), there were no private ENT clinics operating within the region, nor were digital healthcare providers available. Thus, the participating centers represented all available healthcare options for peritonsillitis in the region, with the exception of the three non-participating PHCCs. In Region Kronoberg, patients contacting their PHCC are first evaluated by a triage nurse via telephone, who determines whether a physician’s consultation is required. Each physician visit is assigned a diagnosis code according to the 10th revision of the International Statistical Classification of Diseases and Related Health Problems (ICD-10) or its modified Swedish version (KSH97-P) [23].

### Data extraction

Routine healthcare data from 2012 to 2016 were extracted retrospectively from the comprehensive electronic medical record (EMR) system (Cambio Cosmic, Cambio Healthcare Systems, Linköping, Sweden), which covers both PHC and hospitals in Region Kronoberg. Visit identification number, date, time, and Swedish personal identification number were used for linkage. For this study, data on patient demographics (age, sex) as well as diagnosis codes, laboratory tests, and prescriptions were included.

First, we selected all episodes of peritonsillitis during the study period, including patients of all ages. An episode was defined as the occurrence of a diagnosis code for peritonsillitis (ICD-10: J36.9) in a PHCC or hospital clinic with at least 30 days having passed since the start of the prior episode. Since ICD-10 does not differentiate between peritonsillar abscess and non-purulent peritonsillar cellulitis, and because the registry lacked clinical assessments, our definition of peritonsillitis encompassed both conditions. Cases where a J36.9 diagnosis occurred on the same day as tonsil surgery (code EMB10) were excluded, as these often reflect earlier diagnoses linked to the procedure. Based on personal communication with ENT specialists, a few acute surgeries (á chaud) are performed each year, but we judged that including these would introduce more bias than excluding them.

Second, we selected all episodes of acute pharyngotonsillitis diagnosed in PHC during the same period. Similarly, a new episode was defined as the occurrence of a diagnosis code for tonsillitis (ICD-10: J03x) or pharyngitis (ICD-10: J02x) with at least 30 days having passed since the start of the prior episode. Codes for sore throat as a symptom (ICD-10: R07) and unspecified upper respiratory infection (ICD-10: J06) were excluded, as they lack the specificity needed to reliably identify pharyngotonsillitis.

Third, all data on rapid antigen detection tests (RADTs) for GAS and point-of-care C-reactive protein (CRP) tests recorded in Region Kronoberg during the study period were extracted for the identified patients. The RADT kit used for GAS detection was the QuickVue Dipstick Strep A immunoassay (Quidel Corporation, San Diego, CA, USA). The CRP test used was the Afinion™ CRP assay (Abbott Laboratories, Abbott Park, Illinois, USA), a rapid test with a range of 5–200 mg/l for quantitatively measuring CRP levels.

Fourth, the relevant antibiotic prescriptions recorded in Region Kronoberg during the study period were extracted. These included phenoxymethylpenicillin (penicillin V), cefadroxil, and clindamycin, recommended in the Swedish guidelines for treating pharyngotonsillitis [12]; amoxicillin, erythromycin, and azithromycin, approved by the Swedish Medical Products Agency for this purpose; and metronidazole, commonly used to treat anaerobic infections.

### Statistical methods

The data were cleaned and analyzed using Excel 2019 (Microsoft, Redmond, WA, USA) and SPSS 27 (IBM, Armonk, NY, USA). Although the dataset includes all recorded cases of peritonsillitis in Region Kronoberg between 2012 and 2016, the region and time frame represent a subset of the broader Swedish healthcare system and a limited time period. Therefore, statistical tests were applied to assess whether observed differences are likely to reflect true differences rather than random variation, and to enable broader generalizability.

Population size for each calendar year was obtained from Statistics Sweden (SCB) [23] and represent the registered population on January 1 of each calendar year. Incidence rates were calculated as the number of episodes per population at risk, expressed per 100,000 inhabitants for peritonsillitis and per 1,000 inhabitants for pharyngotonsillitis.

To compare the occurrence of peritonsillitis relative to pharyngotonsillitis in the population, annual ratios were calculated by dividing the number of peritonsillitis episodes by the number of pharyngotonsillitis episodes diagnosed in the same year. This ecological measure reflects the relative frequency of peritonsillitis in relation to pharyngotonsillitis across calendar years, but does not represent individual-level risk.

Poisson regression models were applied to assess temporal trends. For incidence analyses, the annual number of events was entered as the dependent variable with calendar year as an independent variable, and the log of the population size was used as offset. For the peritonsillitis-to-pharyngotonsillitis ratio, the number of peritonsillitis episodes was modeled with the log of the number of pharyngotonsillitis episodes as offset. Incidence rate ratios (IRR) with 95% confidence intervals (CI) were calculated, representing the relative change per calendar year. Model fit was assessed using Pearson chi-square and deviance statistics divided by degrees of freedom; neither indicated overdispersion, and Poisson regression was therefore considered appropriate. Categorical data were compared with two-sided Pearson Chi square test and with one sample Chi square goodness-of-fit test. For differences in continuous variables between groups, t-tests or Mann-Whitney U tests were used, depending on the distribution of the data. P-values < 0.05 were considered significant.

CRP was described as median (interquartile range; IQR) due to non-normal distribution and a constricted measurement range (5–200 mg/l). In line with clinical practice, CRP level < 5 was regarded as zero. Mann-Whitney U test was used to compare CRP distribution between two independent groups.

## Results

### Patient characteristics

Between 2012 and 2016, a total of 607 peritonsillitis episodes were recorded in Region Kronoberg, corresponding to a mean annual incidence of 64 per 100,000 inhabitants (95% CI 53–76) (Table 1). Of these, 371 cases (61%) were confirmed at the ENT department (“ENT-confirmed”), with a mean incidence of 39 per 100,000 (95% CI 31–49). The incidence of peritonsillitis decreased significantly over the study period (IRR per year 0.92, 95% CI 0.87–0.97; *p* = 0.003), as did the incidence of ENT-confirmed peritonsillitis (IRR 0.90, 95% CI 0.83–0.96; *p* = 0.003).

During the same period, 26,534 pharyngotonsillitis episodes were diagnosed in PHC, corresponding to a mean annual incidence of 28 per 1,000 inhabitants (95% CI 27–29). The incidence of pharyngotonsillitis also decreased significantly over time (IRR 0.89, 95% CI 0.88–0.90; *p* < 0.001).

On average, there were 23 peritonsillitis episodes per 1,000 pharyngotonsillitis episodes (95% CI 19–27), and 14 ENT-confirmed peritonsillitis episodes per 1,000 pharyngotonsillitis episodes (95% CI 11–18). These ecological ratios remained stable throughout the study period (IRR 1.03, 95% CI 0.97–1.08 for all peritonsillitis; IRR 1.00, 95% CI 0.93–1.07 for ENT-confirmed peritonsillitis).

**Table 1:** Peritonsillitis and pharyngotonsillitis incidence, 2012–2016 (with 95% CI)

Year	2012	2013	2014	2015	2016	*p*
Population size	185,887	187,156	189,128	191,369	194,628	
Peritonsillitis episodes (n)	142	125	120	121	99	
Peritonsillitis incidence (per 100,000 inhabitants)	76 (64–90)	67 (56–80)	63 (53–76)	63 (52–76)	51 (41–62)	0.003
ENT-confirmed peritonsillitis episodes	90	83	65	77	56	
ENT-confirmed peritonsillitis incidence (per 100,000 inhabitants)	48 (39–60)	44 (35–55)	34 (27–44)	40 (32–50)	29 (22–37)	0.003
PHC pharyngotonsillitis episodes (n)	7,124	5,576	4,530	4,233	5,071	
Pharyngotonsillitis incidence (per 1,000 inhabitants)	38 (37–39)	30 (29–31)	24 (23–25)	22 (21–23)	26 (25–27)	< 0.001
Peritonsillitis per 1,000 pharyngotonsillitis	20 (17–23)	22 (19–27)	26 (22–32)	29 (24–34)	20 (16–24)	0.3
ENT-confirmed peritonsillitis per 1,000 pharyngotonsillitis	13 (10–16)	15 (12–18)	14 (11–18)	18 (14–23)	11 (8–14)	0.9

Annual incidence ratios are presented per 100,000 inhabitants (peritonsillitis) and per 1,000 inhabitants (pharyngotonsillitis), with 95% confidence intervals (CI) based on the Poisson distribution. Ecological ratios are expressed as the number of peritonsillitis episodes per 1,000 pharyngotonsillitis episodes in the population during the same year. These ratios reflect relative frequency at the population level and should not be interpreted as individual risk estimates. P-values refer to temporal trends tested by Poisson regression, reported as incidence rate ratios (IRR) per calendar year

*Abbreviations: ENT *otorhinolaryngology (ear, nose and throat) department, *PHC *primary health care, *CI *confidence interval

The 607 episodes of peritonsillitis occurred in 555 unique patients, the majority of whom (92%) experienced only a single episode during the study period. Of the remainder, 37 patients (7%) had two episodes, 6 patients (1%) had three, and 1 patient (0.2%) experienced four episodes.

Peritonsillitis incidence varied by age, peaking in late adolescence and remaining low in children and older adults (Table 2), with no gender differences within age groups. No significant difference in incidence was found across the months (df = 11, *p* = 0.59).

Compared with patients with pharyngotonsillitis, those with peritonsillitis were generally older, more often female, less frequently prescribed antibiotics on the day of diagnosis, and less likely to undergo point-of-care testing (Table 3). Among those tested, the proportion of positive RADTs was markedly lower in peritonsillitis, while the median CRP value was nearly three times higher. Recurrent pharyngotonsillitis was uncommon in both groups but slightly less frequent in peritonsillitis. Additional characteristics are presented in Table 3.

**Table 2:** Incidence of peritonsillitis in relation to age and gender

Age (years)	Number of peritonsillitis cases (2012–2016)			Average population per year (2012–2016)			Annual incidence per 100,000 inhabitants (95% CI)			*p*
	Female	Male	In total	Female	Male	In total	Female	Male	In total	
0–12	18	16	34	13,960	14,710	28,670	26 (15–41)	22 (12–35)	24 (16–33)	0.6
13–14	7	7	14	1,985	2,104	4,089	71 (28–145)	67 (28–137)	68 (37–115)	0.9
15–19	57	67	124	5,194	5,667	10,861	219 (166–284)	236 (183–300)	228 (190–272)	0.7
20–29	85	71	156	12,022	13,407	25,429	141 (113–175)	106 (83–134)	123 (104–144)	0.07
30–39	57	57	114	10,621	11,588	22,210	107 (81–139)	98 (75–127)	103 (85–123)	0.6
40–49	33	23	56	11,845	12,486	24,330	56 (38–78)	37 (23–55)	46 (35–60)	0.1
50–59	19	19	38	11,126	11,546	22,672	34 (21–53)	33 (20–51)	34 (24–46)	0.9
60–69	25	15	40	11,543	11,885	23,429	43 (28–64)	25 (14–42)	34 (24–47)	0.09
70+	20	11	31	15,362	12,581	27,943	26 (16–40)	17 (9–31)	22 (15–31)	0.3

The table shows the total number of peritonsillitis cases over 2012–2016 (due to relatively small counts). Annual incidence rates per 100,000 inhabitants were calculated using the average number of cases per year and the average population during 2012–2016. *P*-values for comparisons between females and males within each age group were calculated using the exact Poisson method. 95% CIs are based on the exact Poisson distribution

*Abbreviations: CI *confidence interval

**Table 3:** Characteristics of patients with diagnosed peritonsillitis and pharyngotonsillitis, respectively, during 2012–2016

Characteristic, *n* (%)	All episodes of peritonsillitis (*n* = 607)	All episodes of pharyngotonsillitis (*n* = 26,534)	*p*
Age, years, median (IQR)	28 (19–43)	20 (9–36)	< 0.001
Female, *n* (%)	321 (53)	11 635 (44)	< 0.001
Season, *n* (%):			
Winter half-year (oct–mar)	270 (45)	12 071 (46)	0.65
Summer half-year (apr–sep)	337 (56)	14 463 (55)	0.65
Antibiotics^1^ on the day of diagnosis, *n* (%)	365 (60)	20 508 (77)	< 0.001
Antibiotics^1^ prior to the day of diagnosis, *n* (%)			
Last 1 day	28 (5)	17 (0.1)	< 0.001
Last 3 days	62 (10)	52 (0.2)	< 0.001
Last 7 days	78 (13)	126 (0.5)	< 0.001
Last 30 days	113 (19)	655 (3)	< 0.001
Previous episodes of pharyngotonsillitis in the last 12 months^2^, *n* (%)			0,039
0	548 (90)	23 013 (87)	
1	52 (9)	2 896 (11)	
2	7 (1)	506 (2)	
3 or more	-	119 (0.4)	
RADT performed, *n* (%)	170 (28)	18 804 (71)	< 0.001
Positive RADTs/Total RADTs, *n* (%)	64 (38)	12 479 (66)	< 0.001
CRP test performed, *n* (%)	178 (29)	10 006 (38)	< 0.001
CRP, mg/L, median (IQR)	76 (39–138)	27 (5–65)	< 0.001

*P*-values are for Chi-square test (categorical variables) and Mann–Whitney U test (continuous variables). Degrees of freedom: age distribution, previous episodes df = 3; binary variables df = 1

*CRP *C-reactive protein, *ENT *Ear Nose Throat, *RADT *Rapid antigen detection test for Group A Streptococci

^1^ Antibiotics with effect on throat pathogens (see Methods section)

^2^ Excluding the last 30 days, as any diagnosis of pharyngotonsillitis within this period may pertain to the current episode (see Methods section)

^3^ At the day of diagnosis. RADTs were only available in PHC

^4^ At the day of diagnosis. CRP test refers to point-of-care tests in PHC

### Patient pathways

#### First contact

**Fig. 1:**
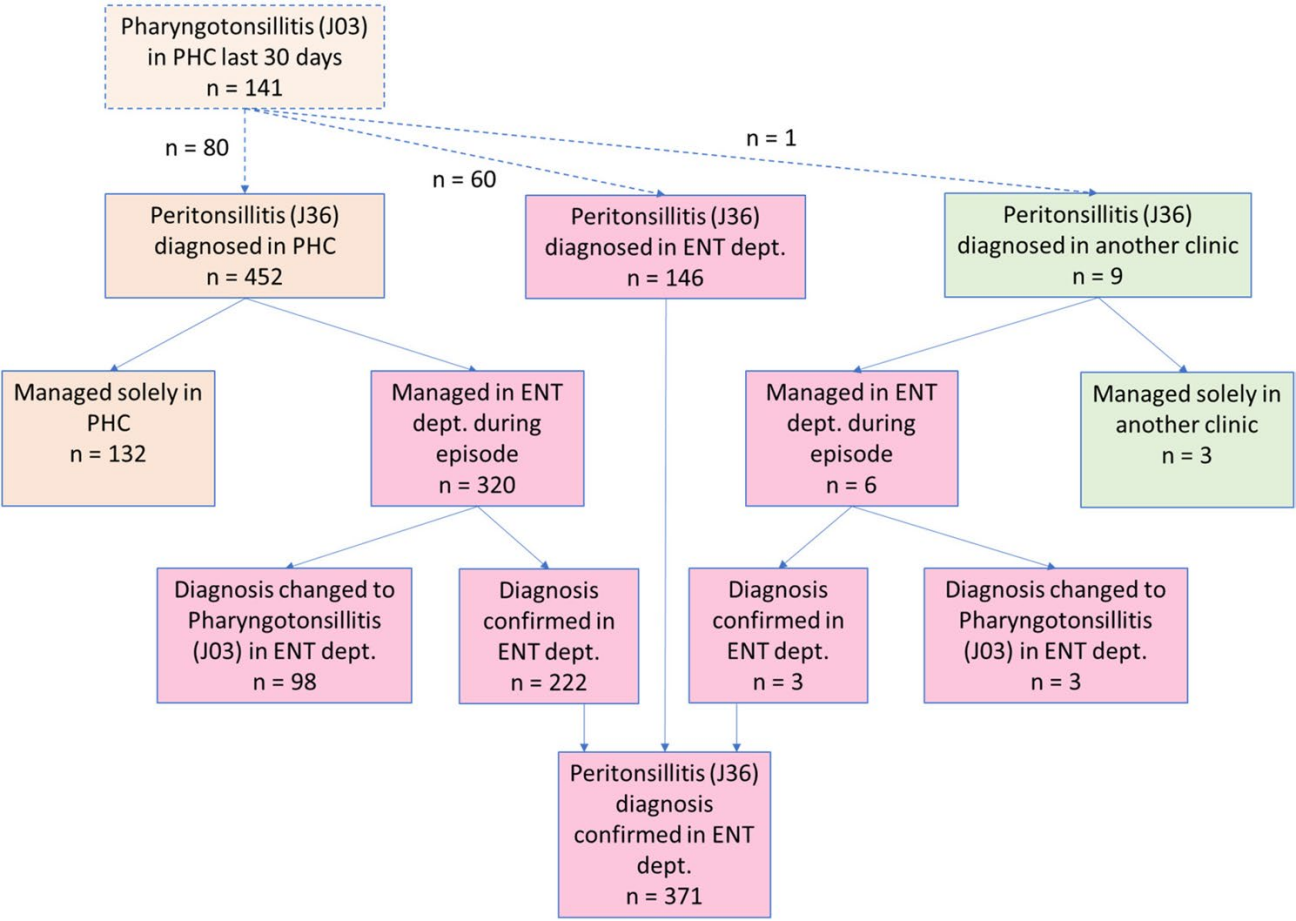
Care pathways of 607 patients diagnosed with peritonsillitis either in PHC (*n* = 452), ENT department (*n* = 146) or another hospital clinic (*n* = 9)

Of the 607 episodes of peritonsillitis, 452 (74%) were first diagnosed in PHC, 146 (24%) in the ENT department, and 9 (1.5%) in other hospital clinics (Fig. 1). Among the 452 episodes diagnosed in PHC, 80 (15%) were preceded by a diagnosis of pharyngotonsillitis in PHC within the last 30 days. The corresponding numbers were 60 out of 146 (41%) for patients first diagnosed in the ENT department and 1 out of 9 (11%) for patients in other hospital clinics. In total, 513 of 607 episodes (85%) involved initial management in PHC, either with peritonsillitis or with pharyngotonsillitis within 30 days prior to peritonsillitis.

#### Transfer from PHC to ENT

Among patients diagnosed with peritonsillitis in PHC, 132 out of 452 (29%) were managed solely within PHC, whereas 320 (71%) were also managed in the ENT department during the same episode. Of these 320 patients, 222 (69%) were assigned a diagnosis code for peritonsillitis at the ENT department, and the remainder were diagnosed with pharyngotonsillitis only. Of the 9 patients initially diagnosed in other hospital clinics, 3 later received a peritonsillitis diagnosis in the ENT department. Overall, 371 out of 607 episodes (61%) were confirmed as peritonsillitis in the ENT department.

#### Timing of ENT confirmation

Of the 222 episodes diagnosed with peritonsillitis in PHC and subsequently confirmed at the ENT department, 196 (88%) were confirmed on the same day, and 217 (98%) were confirmed on the same day or the following day. Only 5 episodes (2%) were confirmed later, with intervals ranging from 3 to 15 days.

#### Pharyngotonsillitis prior to peritonsillitis

**Fig. 2:**
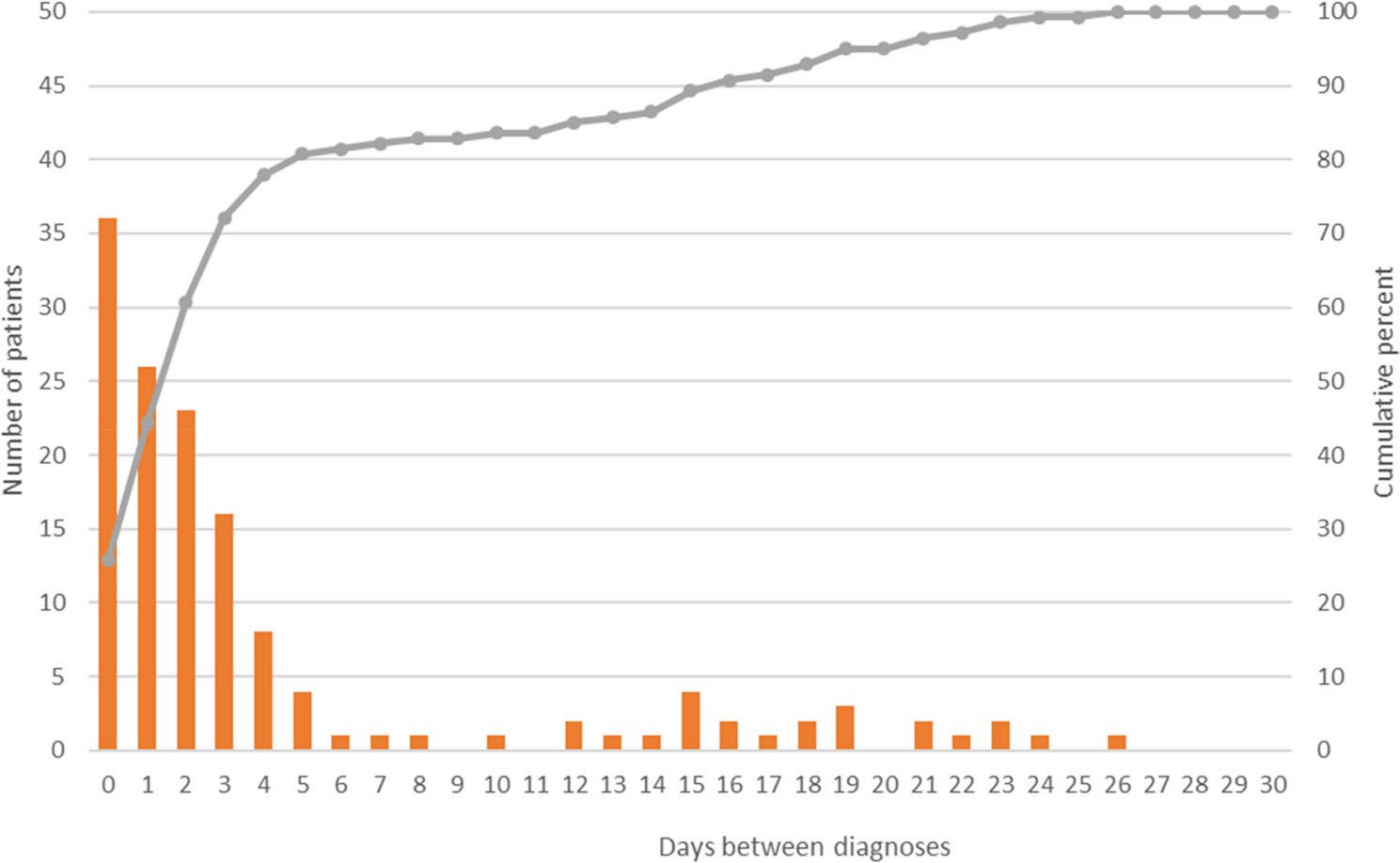
Number of days between the diagnosis of pharyngotonsillitis in PHC and the subsequent diagnosis of peritonsillitis in PHC or a hospital (*n* = 141), limited to peritonsillitis cases occuring within 30 days

A total of 141 out of 607 patients (23%) received a diagnosis of pharyngotonsillitis in PHC within 30 days prior to peritonsillitis (Fig. 1). The median interval between the two diagnoses was 2 days (IQR 0–4) (Fig. 2). Although the maximum interval observed was 26 days, most cases were diagnosed within a few days. When restricting the analysis to the 371 ENT-confirmed cases, 106 patients (29%) had a prior pharyngotonsillitis diagnosis. Antibiotics were prescribed in 96 of 141 patients (68%). The most commonly used agent was phenoxymethylpenicillin (*n* = 57), followed by clindamycin (*n* = 37), amoxicillin (*n* = 1), and cefadroxil (*n* = 1). Excluding the 38 patients diagnosed with pharyngotonsillitis and peritonsillitis on the same day, the antibiotic prescription rate was 77 of 103 patients (75%).

## Discussion

### Main findings

In this population-based study on routine healthcare data, the mean annual incidence of peritonsillitis was 64 episodes per 100,000 inhabitants and decreased significantly over the study period. The highest incidence was observed in adolescents (228 episodes per 100,000). No significant sex differences were found across any age groups. Most patients (74%) were initially diagnosed in PHC. When patients who had sought care for pharyngotonsillitis within the preceding 30 days were also included, PHC accounted for the initial management in 85% of all cases. Overall, precedent pharyngotonsillitis was recorded in 23% of peritonsillitis cases (29% when restricting the denominator to ENT-confirmed cases only). When present, progression to peritonsillitis was rapid, with a median time to diagnosis of two days.

### Meaning of the main findings

#### Patient characteristics

The average annual incidence of peritonsillitis of 64 cases per 100,000 inhabitants was higher than previously reported in Scandinavia (19–42 cases per 100,000 inhabitants) [1–3]. However, these studies reported peritonsillar abscess exclusively from ENT clinics, whereas our findings encompass all conditions diagnosed as peritonsillitis, both in PHC and hospitals. Due to the nature of our register data it is impossible to estimate the proportions of peritonsillar abscess and peritonsillar cellulitis, but in a previous Swedish study on patients at an ENT clinic, using a medical file review, cellulitis constituted 27% of all cases coded as J36 [24]. In our study, the incidence of ENT-confirmed peritonsillitis was 39 cases per 100,000 inhabitants, which is very close to the previous reports and markedly lower than 64. Still, it is hard to deduce if this points to a misclassification of pharyngotonsillitis in PHC, or whether ENT physicians are more reluctant than PHC physicians to classify cellulitis as peritonsillitis.

The parallel decrease in incidence of both peritonsillitis and pharyngotonsillitis over 2012–2016 provides ecological support for the proposed connection between these conditions. The trend suggests that fluctuations in pharyngotonsillitis occurrence are reflected in peritonsillitis at the population level. This observation strengthens the hypothesis that peritonsillitis often arises as a complication of pharyngotonsillitis [5, 12]. The reasons behind the overall decline remain speculative, potentially involving changes in healthcare-seeking behavior, diagnostic practices, or population-level exposure to streptococcal infections. However, the study period was relatively short, and incidence rates have subsequently increased following the COVID-19 pandemic [4], underlining the need for longer-term surveillance.

Peritonsillitis was more common in adolescents and young adults, and exhibited no seasonal variation, which is in line with previous studies [1, 2]. In contrast, we found no gender differences in any age group [1, 2, 6]. RADT for GAS was used in 28% of the patients but was only positive in 38% of these cases, pointing to additional aetiologies and showing that negative rapid tests cannot rule out abscess development [1, 9, 10]. Recurrent episodes of diagnosed pharyngotonsillitis during the last year was scarce (10%) and only 1% had more than one episode.

Compared with all diagnosed episodes of pharyngotonsillitis during the study period, patients with peritonsillitis were older, more often of female gender and had a higher median CRP value. However, none of these traits were discriminate enough to hold any predictive significance when facing a patient with a sore throat.

#### Patient pathways

PHC was the starting point for most patients. Of all patients diagnosed with peritonsillitis in the registry, 74% received their diagnosis in PHC. If also considering those patients with a precedent pharyngotonsillitis, of which most advanced to a peritonsillitis diagnosis within a few days, 85% had PHC as their first contact. This aligns with a previous study, in which 83% of the patients with diagnosed peritonsillar abscess at an ENT clinic self-reported a prior contact with PHC [3]. Interestingly, not all patients initially diagnosed in PHC were subsequently referred to ENT; only 71% eventually received ENT evaluation. The reasons for remaining in PHC are not captured in the registry, but it is plausible that these patients had a milder clinical presentation or represented cases of diagnostic misclassification, such as severe pharyngotonsillitis. Alternatively, the managing physician may have consulted an ENT specialist by phone or followed established treatment protocols without referral. Without detailed medical record review, the exact factors influencing referral decisions remain speculative.

Peritonsillitis is frequently considered a complication of pharyngotonsillitis; however, only 23% of patients in our cohort had been assigned such a diagnosis within the preceding month. The true incidence of pharyngotonsillitis episodes in the population is likely higher, as some individuals may have managed their symptoms exclusively through self-care without seeking medical consultation. Findings from previous investigations at ENT clinics have varied: one study reported that merely 17% of patients recalled a sore throat within the past three months [3], whereas another, examining hospitalized patients with peritonsillar abscess, found that 76% reported a preceding sore throat [13].

Among the 23% patients diagnosed with precedent pharyngotonsillitis in PHC (29% among patients with an ENT-verified diagnosis), most proceeded to peritonsillitis within a few days, in line with previous findings [14], illustrating the rapid progress of peritonsillitis once symptoms start to occur. Among these, some were probably exhibiting symptoms of peritonsillitis rather than of pharyngotonsillitis, as 27% received a peritonsillitis diagnosis the same day. A medical file review might have elucidated this, but it is plausible that some PHC physicians also felt more comfortable using the ICD-10 code for pharyngotonsillitis, rather than for peritonsillitis, when in doubt about the condition or in wait for a ENT-confirmation. Most (75%) of patients diagnosed with pharyngotonsillitis 1–30 days prior to their peritonsillitis received antibiotics, showing that the progress proceeded despite treatment [14], or that the treatment was initiated too late to affect the outcome. This aligns with a retrospective study of hospitalized patients, where 66% of the patients developed peritonsillar abscess despite prior antibiotic therapy [13].

Put differently, 77% of the patients in this study presented with peritonsillitis at their initial visit without a prior consultation for sore throat, which is higher than the 54% reported in a previous Swedish study [3] and the 68% reported in a study from United Kingdom [14]. It implies that few patients show warning signs, and that healthcare personnel must always be alert of symptoms of peritonsillitis.

### Strengths and limitations

A strength of this study is that it presents an almost complete register of routine population data from a Swedish healthcare region during five consecutive years. Originally extracted for another project, the data is a bit old; on the other hand, it mirrors pre-pandemic management of sore throat and is therefore unaffected by altered patterns of prevalence, management and healthcare attendance. In the future, our group plan to do an updated and more comprehensive study, including more regions in Sweden, to study the effect of the Covid 19-pandemic in particular. Though throat culture results were available in the registry, aetiological description was beyond the scope of this project and has more recently been explored and published by others [4, 8–10]. Nor were we able to examine predictive factors, as this would require clinical data from a medical file review of cases and controls.

#### Methods discussion according to RECORD

A third of the ICD 10-codes for peritonsillitis from PHC were altered to pharyngotonsillitis at the ENT department. The meaning of this is not certain, but a feasible explanation is that the ENT physician did not agree with the PHC physician regarding diagnosis. This diagnostic uncertainty in PHC agrees with earlier studies showing that it is difficult to separate early peritonsillitis from acute tonsillitis based on clinical signs alone. Teppo et al. found that while GP diagnostics had excellent sensitivity with no missed cases, the diagnostic specificity was poor, with more than half of the referred patients having a false-positive diagnosis [25]. However, recent data from Voruz et al. shows that making a reliable diagnosis from a physical examination alone is very challenging, even for ENT specialists [26]. This suggests that the differences in coding between primary and secondary care are in part due to diagnostic limitations, rather than just a lack of competence in PHC. The differences could also be due to coding habits, where suspected cases in PHC receive the code of the confirmed condition, or a reluctance among ENT physicians to use the J36 code for cellulitis, though a previous ENT study reported that cellulitis accounted for 27% of J36 diagnoses [24]. Nonetheless, the lack of medical file review data means that key clinical diagnostic criteria, such as the presence of trismus (restricted mouth opening), could not be verified. This makes it difficult to rule out a classification bias and should therefore call for a cautious interpretation of the data. There were no economical incentives for coding, and neither were any coding practices changed during the study period.

Our definition of an episode is a pragmatic construction to be able to relate to registry data and does not necessarily reflect the clinical picture. Therefore, some residual uncertainty is inevitable. At the same time, as most patients only experienced one episode of peritonsillitis, the problem is of limited nature. It is somewhat different for patients with pharyngotonsillitis, as recurrency is more frequent here, but also in this group was only a small proportion with more than one episode. We chose a 30-day cut-off somewhat arbitrarily, but it is a commonly used definition of recurrence for infections and was determined a priori.

A limitation of this study is that three of the region’s 34 PHCCs did not participate. Since the incidence rates were calculated using the total population of Region Kronoberg as the denominator, the reported incidence may be slightly underestimated. However, this underestimation is unlikely to affect the overall conclusions.

We believe that the register caught all episodes of peritonsillitis and pharyngotonsillitis from the 31 out of 34 PHCCs that participated. However, with only 60% of patients with peritonsillitis receiving antibiotics on the day of diagnosis, one must suspect some missing data. As many of these prescriptions ought to be from the ENT department, there might have been some error in the retrieval of hospital prescriptions.

We lacked information on the clinical rationale for performing CRP or RADT when diagnosing pharyngotonsillitis, as well as on the decision-making underlying antibiotic prescribing. This introduces a considerable risk of indication bias, since patients presenting with more severe symptoms are more likely to be tested or treated, which could distort the results. Nevertheless, even under this potential bias, CRP values did not sufficiently discriminate between patients with peritonsillitis and those with pharyngotonsillitis, thereby reinforcing the conclusion that CRP has limited predictive value for peritonsillitis among patients presenting with sore throat.

In this study, we intentionally focused on point-of-care tools readily available to the PHC clinician during the actual consultation, as these directly influence immediate management decisions. Consequently, routine microbiological culture data was not investigated. Such culture data often carries methodological limitations regarding polymicrobial infections compared to modern PCR diagnostics, and the registry lacked crucial clinical context, such as symptom duration and disease severity, which would be necessary to accurately interpret culture results.

The database lacked detailed clinical follow-up data for patients diagnosed with peritonsillitis in PHC who were not referred to an ENT department. Consequently, we were unable to assess their prognosis, symptom duration, or disease severity, which remains a limitation of using routine registry data.

## Conclusions

Peritonsillitis predominantly affected adolescents, with no sex differences, and commonly presented without a recorded preceding sore throat. Most episodes were first diagnosed and initially managed in PHC, highlighting the central role of PHC in identifying established or developing cases. Clinical vigilance is essential, as clinical features, including point-of-care tests, provide limited predictive value and diagnosis relies largely on clinical evaluation. In episodes following recent pharyngotonsillitis, progression was rapid, underscoring the need for careful evaluation of patients with worsening symptoms.

## Data Availability

The datasets used and/or analysed during the current study are available from the corresponding author on reasonable request.
